# Experiences of everyday health support and healthcare among settled young people who arrived as unaccompanied refugees in Norway

**DOI:** 10.1080/17482631.2026.2705751

**Published:** 2026-07-23

**Authors:** Gjertrud Moe, Linn Okkenhaug Getz, Bente Prytz Mjølstad, Borgunn Ytterhus

**Affiliations:** a Department of Public Health and Nursing, General Practice Research Unit (AFE Trondheim), NTNU, Trondheim, Norway

**Keywords:** Health, qualitative, recognition, GP, unaccompanied refugees, person-centred care

## Abstract

**Purpose:**

When young unaccompanied refugees are settled in a local municipality, they are expected to gradually take responsibility for organizing their everyday lives, including accessing public services. This study aims to examine these young people's experiences of health-related support and primary healthcare in everyday life when settled in Norway.

**Method:**

In-depth interviews with nineteen young unaccompanied refugees (18–25 years) from Afghanistan, Eritrea, and Syria. The interviews were analysed using interpretive phenomenology. Theoretically, Axel Honneth's philosophy of recognition is applied.

**Results:**

The young unaccompanied refugees use their agency to manage daily life, support and care for family abroad, and attend school; however, the absence of primary relations loving forms made them vulnerable in terms of self-confidence, as described in Honneth's taxonomy. They emphasized that professionals must integrate care and recognition into routine services to maintain their well-being and dignity. Teachers and school nurses offered relational continuity and meaningful support, but the participants felt that their concerns were insufficiently acknowledged by GPs when presenting indistinct symptoms such as headaches or stomachaches. Still, most of the young participants reported high-quality care from their GP when dealing with acute or clearly defined medical issues.

**Conclusion:**

The young refugees revealed both agency and vulnerability. They also expressed unmet needs for recognition, particularly within their primary relationships. In this context, teachers and school nurses provided availability, continuity and person-centred care, enabling participants to feel recognized and able to manage everyday life. Although GPs encounter them less frequently and within stricter time constraints, their role remains pivotal. Strengthening GPs' capacity to recognize young refugee patients as whole persons, including their need for recognition and their emotional vulnerability, represents an important area for improvement.

## 1. Introduction

The aim of this article is to examine young settled unaccompanied refugees' experiences of receiving healthcare services and health-related support in everyday life. Refugees represent one group among migrants. While migrants in general are reported to have better health than their native counterparts in Norway on arrival, their health condition tends to decline the longer they reside in Norway (Spilker et al., 2017). Young unaccompanied refugees are considered particularly vulnerable because they are by definition “separated from both parents and are not being cared for by an adult who, by law or custom, is responsible to do so” (UNHCR, 1994). Their experiences before and during their flight, together with the challenges of resettling in a new country, place considerable demands on their health and well-being (Oberg & Sharma, 2023). Systematic reviews examining the mental health of unaccompanied refugees residing in European countries have shown that they are at higher risk than their peers are of developing anxiety, depression and posttraumatic stress disorders (PTSDs) (Daniel-Calveras et al., 2022; Kien et al., 2019). Young unaccompanied refugees also report lower quality of life than the general population due to post-migration stressors and mental distress (Garbade et al., 2025). Young people in late modern society generally have a more protracted transition into adulthood than in the past (Osgood et al., 2010), but this is not the situation for young unaccompanied residents. In many ways, they face an accelerated transition into adulthood (Sirriyeh & Ní Raghallaigh, 2018), where they need to cope with challenges concerning education, work, living arrangements, and family (Garvik et al., 2016). Despite that, studies on resilience (Brook & Ottemöller, 2020; Rodriguez & Dobler, 2021) and coping strategies (Behrendt et al., 2023; Ni Raghallaigh & Gilligan, 2010; Scott et al., 2022) point out that unaccompanied refugees possess important resources and strengths. A quantitative cross-sectional study of unaccompanied refugee minors in Norway who had been granted residence permits examined resilience and protective factors in comparison with Norwegian adolescents, as well as associations between resilience factors and positive developmental outcomes (Heimli et al., 2024). Overall, the unaccompanied minors scored lower than their Norwegian peers on most of the resilience subscales. Interestingly, unaccompanied female minors demonstrated more resilience factors in certain life domains than males, contrasting with previous studies reporting higher levels of mental health problems among girls (Derluyn et al., 2009; Hodes et al., 2008; Höhne et al., 2023). Frequent contact with family members in the country of origin was associated with more positive development outcomes (Heimli et al., 2024). Although many unaccompanied minors have experienced stressful or traumatic events associated with war, violence and conflict (WHO, 2022), as well as the loss of family members and persecution (Hopkins & Hill, 2008), such experiences do not necessarily result in adverse outcomes (Ni Raghallaigh & Gilligan, 2010). Similarly, a longitudinal study of young unaccompanied refugees resettling in Norway found that, despite experiencing mental health challenges, many remained strongly motivated to build a better future and pursue their aspirations (Andersson & Øverlien, 2023). It must be remembered, though, that refugees are a heterogeneous group with a variety of health needs (WHO, 2022). Nevertheless, migrants generally use the health services less than or equal to those of the majority population (Sarría-Santamera et al., 2016), and young refugees use mental health services less than native peers (Björkenstam et al., 2022; Gubi et al., 2022). Research on young unaccompanied refugees' experience of primary healthcare is limited (Berg et al., 2020). However, previous research has identified several challenges when it comes to providing primary healthcare to immigrants and refugees (Brandenberger et al., 2019; Småland Goth & Berg, 2011). Some studies address issues with trust and the importance of building trust relationships (Feldmann et al., 2007; Haj-Younes et al., 2021; Moe et al., 2025), others report challenging communication due to language barriers (Goth et al., 2010; Harris et al., 2020; Rothlind et al., 2018), mismatched expectations and different understanding of health and the healthcare system (Goth et al., 2010; Harris et al., 2020) as obstacles to equitable healthcare. Additionally, migrants report being less satisfied with their GP compared to the general population (Detollenaere et al., 2018; Lien et al., 2008). A study that focused on young unaccompanied refugees in particular reports that they did not feel that they received the help and support they expected and were not taken seriously when seeking healthcare with complaints such as headaches and stomachaches (Zijlstra et al., 2019).

### 1.2. The Norwegian context

Norway is a geographically extensive country with a well-spread population of 5.4 million. Among these, 300,000 individuals have a refugee background (SSB, 2025), including 12,000 who have arrived in Norway as unaccompanied minors. They are predominantly male (82%), mostly originating from Afghanistan, followed by Eritrea and Somalia. More recent arrivals include refugees from Syria and Ukraine, each comprising approximately 8% of the total group (Kirkeberg, 2025). A significant influx of unaccompanied refugee minors occurred during the refugee wave around 2015 (Kirkeberg et al., 2022). Since 2022, Ukrainian minors have represented the largest group among unaccompanied refugee children. As of today, most individuals who were granted residences as unaccompanied minors have reached adulthood and have been living in Norway for several years (Kirkeberg, 2025). This article, therefore, focuses on the 18–25 years of age group to inquire into their experiences of receiving support to promote their health in everyday life after resettling in Norway.

When unaccompanied minors are granted a residence permit in Norway, the local authorities have a legal duty to assess their needs and provide services that constitute beneficial conditions for child development and integration. Such services include housing, practical help, education, and care (Kommunenes Sentralforbund, 2016). Young unaccompanied refugees between 18 and 25 years may continue to receive child welfare services if needed, and such services are called *child welfare aftercare* in the Norwegian welfare system (Barnevernloven, 2024). However, reports show that aftercare typically ends by the age of 20 (Garvik et al., 2016; Lidén et al., 2020). When aftercare ends, the young unaccompanied refugees are expected to be more independent. However, reports show that they may feel abandoned and need more help in terms of support and guidance when the aftercare comes to an end (Garvik et al., 2016).

The service provider responsible for aftercare differs from one local authority to the next (Lidén et al., 2020), and the local authorities have discretion to decide their organization (Kommunenes Sentralforbund, 2016). In our study, these service providers were named “primary contacts”. They were employed within the social welfare sector and were expected to provide support and guidance, but did not hold a coordinating role in relation to other services working with young unaccompanied refugees. Having a social network and receiving support are of significant value for a person's health and constitute a prerequisite for experiencing well-being and a meaningful life (Mæland, 2020, pp. 104–109). The young refugees have a legal right to access school (Kommunenes Sentralforbund, 2016). However, there is no national guidance on how schooling should be organized specifically for young, settled, and unaccompanied refugees (Lidén et al., 2020). Nevertheless, according to Section 9 A-2 of the Education Act (1999), schools are mandated to serve as a health-promoting arena for all students.

Young unaccompanied refugees have limited access to care relationships. For this reason, it is important to examine the role primary healthcare plays when it comes to the needs these young individuals have for help and support with issues related to health and well-being. This study aims to answer the following research questions:

− How do young settled unaccompanied refugees (18–25 years of age) experience health-related support and healthcare in everyday life, and who are their main helpers when it comes to health and well-being?
− What role do GPs play in relation to young settled unaccompanied refugees' (18–25 years) experiences of help and support with health-related issues?

### 1.3. Norwegian primary healthcare for young unaccompanied refugees

In this article, primary healthcare service refers to GPs and public health nurses, as they are the main providers of health services for young people in Norway. The right of every citizen to be assigned a regular GP also applies to refugees when they become residents of a Norwegian municipality. The regular GPs are responsible for all general medical care for their patients, including acute medical assistance during opening hours. The GP services are based on open access, but appointments are prioritized and scheduled based on the urgency of the problem. Moreover, GPs serve as gatekeepers to specialist healthcare, and referral from one's GP is necessary to gain access to hospital services, except in urgent cases. Specialist services include both mental and somatic services and are universally available. GP visits and specialist services are free of charge for young people under 16 years, while older individuals pay a limited fee for each visit, up to an exemption card limit (Forskrift om fastlegeordning i kommunene, 2012).

*Public health nurses* are a statutory healthcare service provided free of charge for the 0–20 age group (Forskrift om helsestasjons- og skolehelsetjenesten, 2018). They operate within interdisciplinary teams across child health centres (0–5 years), school health services (6–20 years, or up to the pupils' age, which may extend to 25 years for those with a refugee background), and youth health centres. While youth health centres typically serve individuals up to age 20, some municipalities extend the age limit to 25 (Norwegian Directorate of Health, 2023). Public health nurses are tasked with systematically promoting good health and preventing disease among children, adolescents and their families at the individual, group and societal levels. Their scope of practice includes initiating health-related interventions and referring individuals to other healthcare services when needed. However, they are not authorized to initiate medical treatment, with the exception of prescribing contraception (Forskrift om nasjonal retningslinje for helsesykepleierutdanning, 2021). Since the group we are studying primarily accesses the service provided by public health nurses through the upper secondary school system, the term *school nurse* will be used throughout this paper.

### 1.4. Theory of recognition

In this study, we use Honneth's theory of recognition as an analytical framework to explore essential aspects of young unaccompanied refugees' experiences of health-related support and needs in everyday life (2005; 2003). Recognition is a fundamental condition for human beings' relationships with themselves and their surroundings and consequently achieves healthy identities. We argue that recognition is a crucial dimension that educational and primary healthcare personnel must engage with to support the development of healthy identities among young settled unaccompanied refugees, which is necessary for achieving meaningful inclusion processes. These young individuals, who transition into adulthood, are especially vulnerable to negative health outcomes because of their past experiences and the absence of close family members during their formative years (Svendsen et al., 2018). Young settled unaccompanied refugees report that they create healthy identities through meaningful activities that provide recognition (Moe & Ytterhus, 2022). A closer look at recognition might help us understand why young unaccompanied refugees act like they do. This may introduce an important and motivating perspective for professionals who provide health-related services to this group.

Honneth posits that recognition must be seen as the foundation for the moral development of society and for avoiding disrespect, which could generate social conflicts and disintegration (Honneth, 2003). Honneth describes three different levels of developing moral status, which are different spheres within social life praxis: (i) *primary relationships*, (ii) *legal relations* and (iii) *community of value*. Each level is constituted by forms of intersubjective recognition, which Honneth identifies as love, rights and solidarity (Honneth, 2005, p. 129). Together, these three forms of recognition constitute the basis for individuals to attain and maintain self-confidence, self-respect and self-esteem.

According to Honneth, *primary relationships*, such as those formed between parents and children or close friends, play a vital role in the development of self-confidence. These relationships, referred to as the love form, are characterized by particularistic care and emotional attachment within small, intimate groups (Honneth, 2005). *Legal relations* pertain to rights and recognize individuals as capable of taking part in a civil society. The *rights* form, a fundamental premise for individuals' self-respect, concerns equality and equal rights to all human beings (Honneth, 2005). The *community of value,* which is the solidarity form, refers to an individual's relation to groups and wider society, where they are recognized for their unique traits and abilities, contributing to their development of self-esteem. Honneth (2005) claims that recognition is needed on all three levels to develop a healthy identity.

Everyday life encompasses both trivial and special aspects within various institutional and substantial fields. Here, the trivial refers to everyday rhythm and continuity, whereas special refers to exclusivity, positive and negative interruptions and “happenings” (Gullestad, 1992), which contrast with the usual patterns.

When experiencing stress or a personal crisis, an individual will turn to primary relationships for support and care (Houston & Dolan, 2008). As the young settled unaccompanied refugees have limited access to primary relationships, it is important to examine their legal relations and community of value. Public education, health and welfare services represent these levels, and young unaccompanied refugees are dependent on these relations when they need help and support with health-related issues.

## 2. Method

### 2.1. Study design and participant recruitment

This study uses a cross-sectional design based on an applied interpretive phenomenological methodology (Brinkmann & Kvale, 2015). Data were obtained through individual semi-structured life-form interviews (Andenæs, 1991) with young settled unaccompanied refugees living in both urban and rural municipalities in Norway. The rationale behind applying a qualitative approach is to capture the complexities of people's lives and experiences from the participant's point of view (Brinkmann & Kvale, 2015). The following inclusion criteria were used for a purposeful sampling strategy for recruiting young settled unaccompanied refugees:

− 18–25 years old at the time of the study's interviews
− Fled from the country of origin without a parent or caregiver
− Settled as a resident in a Norwegian municipality

In total, we succeeded in recruiting nineteen participants from three different Norwegian municipalities with inhabitants ranging from 7000 to 200,000. Each municipality assigned contact persons to distribute information leaflets and formal information letters. Seventeen participants were recruited in this manner, while two were recruited through the “snowball method”, which is an extended recruitment approach within qualitative research (Andrews & Vassenden, 2007).

### 2.2. Participants and data generation

The participants' countries of origin were Afghanistan, Eritrea, and Syria, and they arrived in Norway from late 2014–2019. Thirteen arrived as minors, and six came after they were 18. The interviews were carried out in 2020 and 2021, and at that time, the participants had lived in Norway for one to five years. At the time of the interviews, the total number of settled unaccompanied minor refugees in Norway was 71 in 2020 and 117 in 2021 (Statistics Norway, 2026). Their time of residency in Norway ranged from one to seven years. The characteristics of the participants are presented in Table 1.

**Table I.** Description of the sample at the time of interviewing.

Category		Participants
Gender	Male	16
	Female	3
Age	18–25 years of age	19
Residence permit	Permanent	10
	Temporary	7
	ID in doubt	2
School/education^a^	Introductory course^b^	2
	Primary school	5
	Lower/upper secondary school	10
	University	2
Living situation	Single household	13
	Shared housing with service provision	1
	Relatives/boyfriend/girlfriend	3
	Friend/roommate	2

The participants are not presented individually to preserve their anonymity and protect their identity.

^a^ Refers to the school or educational level at which the participants were enrolled at the time of the interview.

^b^ A compulsory introductory course for newly arrived migrants.

Thirteen of the interviews were conducted by the first author, and six were conducted by a researcher on the project team. Before the interviews, the participants were given the option to use an interpreter. All declined, either because they saw themselves or were considered by their primary contact person to have a good command of spoken Norwegian. Another reason was mistrust of the interpreter service, despite its confidentiality obligations. Some feared that interpreters with ties to their country of origin might access information that could lead to reprisals. The interviewers found that the participants generally articulated themselves well. Their decision to manage alone in Norwegian as best they could often resulted in speech that was somewhat minimalistic, pointed and condensed, yet conveyed substantive content. However, many nuances might have been richer if the informants had been free to speak in their mother tongue. Most of the interviews lasted around 50 min (range: 23–85 min). All the interviews were recorded and transcribed verbatim by the first author and researchers on the project team.

### 2.3. Data analysis

We analysed the data using an interpretative phenomenological approach applied from Brinkmann & Kvale's (Brinkmann & Kvale, 2015) recommendations for an analytical procedure. The analysis procedure consisted of continuous back-and-forth between the interview transcriptions and the knowledge building, but included all the steps that Brinkmann and Kvale (2015) described. First, we read through all the transcripts to make sense of the whole. We then read each interview to identify meaning units written as close to the participant's own utterances as possible. In the third step, we compared the meaning units within and across the interviews to analytically construct key themes relevant to the research question. At this stage, we left strict narrative descriptions, and the interpretive turn commenced. The final step was to search for patterns that revealed the essence of the analytical themes. The first author analysed the data completely, whereas parts of it were analysed separately by the fourth author. These two then compared and validated the analysis together. Possible interpretations and categorizations developed through the theoretical lens were discussed, switching back and forth between theory and data. We reached a consensus as a team, where all the authors participated in the later stages of the analytical process. All authors have read and accepted the final version of the article.

### 2.4. Ethical considerations

Following legal regulations for personal protection and ethical approval, the study was approved by the personal data protection services in Norway: the Norwegian Center of Research Data (reference code: 175773). The study was submitted to the Regional Committees for Medical and Health Research Ethics South East, and an evaluation was received (reference code: 66230). All potential participants received written information about the study, which we repeated orally. We emphasized that participation was voluntary, that the data would be processed confidentially, and that personal identifiers and biographical information would be anonymized prior to publication. We obtained informed written consent from all participants before each interview. Extra precautions were taken to ensure relational ethics within the interview by staying calm, accepting silence, and reframing questions when necessary, so that the participants felt safe to address any ambiguities during the interviews.

## 3. Results

By using Honneth's framework of recognition to examine the young settled unaccompanied refugees' experiences of health-related support and help, our analysis arrived at three analytical themes: (i) reversed care relations and support, (ii) need for relational continuity in everyday life, and (iii) ambivalence related to Norwegian GP services.

Some of the participants mentioned language as a challenge when seeking help within the healthcare system, even though they had a good command of oral Norwegian. Language challenges are well established in the literature on young unaccompanied refugees (Cheng et al., 2015; Robertshaw et al., 2017). In this study, we chose to set language aside, not only owing to a lack of relevance, but also to explore psychosocial and relational factors that also warrant attention.

### 3.1. Reversed care relations and support

The young newly settled individuals are physically separated from their parents and other close adults and relatives, who constitute the main pillars in Honneth's loving form, which are within primary relationships. Only three of our participants lived with relatives/boyfriend/girlfriend. However, most of them had developed new friendships after their arrival in Norway. Several expressed that their parents were no longer someone they felt that they could lean on for encouragement, support, and help.

*No, I mean first of all I can’t be sad when I talk to them, I mean, they want me to show my feelings of course, but my dad and my mom, they’re old people, they’re not so very old but like you know, they have had enough [...].* (Female, 25 years old)

Consequently, the responsibility they felt they had for their parents' well-being reversed the caregiving aspects of the parent‒child relationship. Furthermore, for some of the youngest participants who still had a primary contact person at the local authority, this person functioned as support and a substitute caregiver. Two participants said:

*I think she’s the best, yeah…she’s kind, yeah.* (Male, 19 years old)

*Yeah, the contact person is very kind, and she’s very kind to me. […] I trust her.* (Male, 20 years old)

The primary contact person, whom they saw on a regular basis, was generally someone they trusted, whom they could talk to, and who helped them ask for healthcare if they needed it. All the young refugees considered them to be someone they could contact when they were in need of practical help and support, but beyond this, their emotional relationship with the contact persons varied greatly. Nevertheless, looking at the situation of the young unaccompanied residents, they are now living without their family in a new country. Some of them had friends, and they shared a mutual understanding of needing to support each other, but the amount of recognition in the love form was limited. Therefore, in asking for help and support, they were dependent on what Honneth describes as the rights form to a much higher degree than peers living with parents/caregivers.

### 3.2. Need for relational continuity in everyday life

The young participants expressed a need for relational continuity in relation to support and help. In addition to their primary contact persons, *teachers* appeared to be the most significant supportive figures in their everyday lives. Our results show that the teachers referred to were aware of and contributed to this. Norway has free access to education, and all our participants were in the school system, but at different educational levels (see Table I). Teachers were, therefore, adults who saw and communicated with these young residents almost daily.

*The teachers are caring, like…they…they’ve got it, like…they understand how we feel or stuff like that, how things are going for us.* (Male, 20 years old)

The participants described the teachers as caring, showing understanding for their life situation, and seeing and recognizing them. This is also verified when we find that in some cases, the teacher was the one who encouraged them to go and talk to the school nurse when they thought the participants needed help with health-related issues that exceeded their own competencies.

*So like when I go to school, I’m late because I can’t sleep, so I have to drink a lot of coffee to concentrate and a teacher saw me and told her [school nurse] and says she wants to have a talk with me, yeah.* (Male, 24 years)

Even though the teachers were busy, the encounters with them on a daily basis appear to have created a continuity that served as the foundation for reciprocal and sustainable recognition and trust.

*The school nurse* represented a previously unfamiliar profession for most of the study participants. This role did not exist at the schools in their home countries. They described the school nurse as a significant source for their health literacy, representing a more holistic understanding of health than their former understanding. Especially, the term “mental health” was new for several of the participants, but has since become something they acknowledge as important and connected to physical health. Many of the participants clearly expressed that stress-related feelings and negative thoughts affect their body and mind.

*Mental health and physical health. The two are interconnected, yeah. If you have poor mental health, that can affect your physical health too.* (Male, 20 years)

Several of the participants had talked with the school nurse about health-related issues that affected their everyday life. Those who had only one individual meeting described the encounter as a good experience and did not find it necessary to return later, or they did not feel that the school nurse could solve their problems. The participants who met with the school nurse several times referred to the nurse's office as a place where they could talk and felt that they received good advice to cope with health issues in everyday life. They categorize the health issues they talked about with the school nurse as not particularly serious from a medical point of view.

*I have been to the school nurse like once here in […], like. But yeah… it wasn’t so serious, like.* (Male, 19 years)

For more serious health-related problems in need of medical treatment, they were referred to their GP.

### 3.3. Ambivalence related to the Norwegian GP services

When seeking help from the GP, the young unaccompanied refugees had expectations of receiving support and help based on the comprehensive understanding of health that they had learned at the Norwegian introductory courses, through the school nurse and through the support system in the municipality. The participants described how they tried to manage on their own most of the time and that they would turn to healthcare services only if they needed help and support to manage health-related issues they themselves considered to be important enough. Typically, this would include “common” complaints, such as minor physical injuries, headache, stomachache, or a troublesome rash. The young refugees' understanding of the healthcare system was partly rooted in personal experiences from their country of origin and partly in what they had learned about health and healthcare since their arrival in Norway. In general, there were substantial differences regarding their expectations, both with respect to the healthcare system as such and cultural approaches to health and healthcare. Participants from unstable political regimes and low-income countries like Afghanistan tended to have minimal personal experience of healthcare and expressed limited trust in the healthcare system in their country of origin. This led to expressions of gratitude that, as a resident in Norway, one can make an appointment with a GP, trust the healthcare system, feel safe and need not worry about economic expenses.

*I feel very safe. Because I feel that if I were to get ill, for example, then I will receive good care. So, I can go to the doctor’s office, there’s no problem if you have financial difficulties or you don’t get treatment because you don’t have money, and things like that, for example.* (Male, 20 years)

Some of the participants emphasized the doctor as an expert and authority in health matters.

*…doctors are right, you know, in a way.* (Female, 23 years)

*All [doctors] know how they treat people and how serious it is…have gone to school, like, yeah, lots of experience and stuff…so they know better than us.* (Male, 19 years)

Even if the participants were basically positive about the organization of the Norwegian healthcare system and the resources it can provide access to, many of them described how their expectations in relation to the GP services were not met. In particular, newly settled individuals from previously stable middle-income countries like Syria were used to a healthcare system where access to a family doctor was easy. Having to wait days or even weeks to obtain a regular appointment with a Norwegian GP, thereby led to disappointment. One participant described the contrast:

*… you have to wait one week or two weeks…like it’s…[…]… I can’t sleep…In Syria, it is not like that. You talk with the doctor…“okay, I come [to the doctor’s office].”* (Male, 24 years)

The absence of recognition was expressed by participants who expected quick healthcare service from their GPs and were disappointed as they had to wait.

Norway's way of organizing the healthcare service with a regular GP who also has a gatekeeper role for access to specialist healthcare was also new to the participants. Beyond this, they described how their expectations were not only related to biomedical diagnosis and treatment; they also expected to be seen and cared for as whole persons. They expected the GP to be an authority that would acknowledge them as human beings as a basis of trust, an adult to rely on. In terms of Honneth's framework of recognition, the participants seemed to expect the GP to embody the professional norms of the legal form, yet remain capable of shifting towards the more relational stance characteristic of the love form when the situation called for it.

The participants' understanding of medical prescriptions differed. Some participants were positive about the fact that prescribing medication is not always the solution and were critical of the use of medication in their country of origin. However, several participants expected medication and became unhappy because the GPs did not explain why they did not prescribe any medication. If they had been given an explanation, they might not have felt left alone with the responsibility or that their complaint was unimportant. Some even wondered if the doctor had something personal against them, as explained by one participant:

*The doctor, he should understand this because we think “okay, he doesn’t give us medicine, why?” He hates me or like…something.* (Male, 24 years)

Another participant struggled to understand why her GP did not examine her physically. In her home country, it would be routine to perform physical examinations when going to the doctor. As the interview took place during the COVID-19 pandemic, the participants questioned whether this lack of a physical examination might have been related to the hygienic recommendations of health authorities on physical distancing, but the participants still found this difficult to understand. Instead of getting help, she ended up feeling that her GP only saw her as a potential contagion:

*…when we get sick and we see a doctor, a doctor will come close and will touch us and like, will like, will open our mouth and like do a lot of stuff. Would touch us, here I feel like when you go to a doctor you, you’re like, I don’t know, I feel like I’m a virus.* (Female, 25 years)

#### 3.3.1. Absence of human recognition from the GP

In cases where the participants' experience of healthcare was related to acute or specific health needs, such as physical injuries (e.g., a bone fracture), they felt that they received the help they needed. However, when visiting GPs with less tangible symptoms, such as headaches and stomachaches, they often felt that their concerns were not taken seriously. Although they described the GP as friendly, they experienced that the GP lacked interest in them as persons. They were not given sufficient time, the doctor did not ask questions that could clarify the problem and did not show interest in them and their everyday life situation.

*…so he like doesn’t care about me, you know. He doesn’t ask “what do you do? Where do you go? What’s your day like?”, and stuff like that, you know. He doesn’t show any interest, you know…*(Male, 18 years)

When the GP's focus remains solely on symptoms and diagnoses, without inquiry into their everyday life and challenges, the young refugees perceive a disappointing lack of understanding and personal engagement. This experience aligns with what Honneth (2005) describes as a form of disrespect—where individuals feel unseen and not taken seriously, resulting in a perceived absence of recognition. In this context, several participants made a point of how the GP's main advice for various symptoms (e.g., headache and stomachache) appeared to be “drink water”.

*He doesn’t take more than five minutes, like, you know. So he looks at you, like, also like “no, you just have to drink water”. Then I get depressed…* (Male, 18 years)

.*..when we go there, she just says “drink water”. Water is very important. Just drink water. Oh my God!* (Male, 20 years)

In such situations, the young unaccompanied refugees are left without any medical diagnosis or explanation that makes sense to them. They feel that their worries are not really understood and do not know what to do. Nevertheless, some of the participants felt that the GP did not acknowledge them by showing interest in them as individuals and being aware of cultural differences.

Exceptions existed, though. One participant talked about a former GP who recognized him, showed interest in him and asked questions of relevance.

*She was good at talking, “how is it going with you (…)? Are things good at work? Are you going to school, or?” so she gave me lots of alternatives, lots of opportunities to talk about everything that happens in my everyday life.* (Male, 24 years)

This participant perceived his GP as a highly skilled doctor who saw and acknowledged him as a person.

## 4. Discussion

This study provides insight into young settled unaccompanied refugees' experiences of health-related support and primary healthcare in everyday life when settling in Norway. It has three main results: (i) reversed care relations and support, (ii) the need for relational continuity, and (iii) ambivalence related to Norwegian GP services. These three main results will be discussed in the following sections.

### 4.1. Reversed care relations and support between the young unaccompanied refugees and their parents

Although young unaccompanied refugees are often described as a vulnerable group owing to their life situations and experiences, research also shows that they actively apply coping strategies (Behrendt et al., 2023) and demonstrate resilience in navigating new social and institutional contexts (Brook & Ottemöller, 2020). When attempting to provide a picture of the everyday situation for young unaccompanied refugees, our results show that the care relationship with their parents has typically been reversed. The young people are thereby put in a situation where they need to provide care and support for them. The shift in role-content has interesting similarities to children next of kin who have parents with illnesses or who struggle with substance abuse that completely or partially incapacitate them from fulfilling the responsibilities expected in parenthood. They take over the responsibility for household, personal, and emotional tasks from their parents (Järkestig‐Berggren et al., 2019; Kallander, 2020). Although the young participants in our study are not daily caregivers for their parents, the parallels between these groups indicate that everyday challenges arising from limited or lacking recognition in primary relationships may also apply to other groups temporarily separated from such relationships. They experience a lack of “loving forms” (Honneth, 2005), and in some cases, parental demands for support exceed the participants' practical and emotional capacities, becoming an everyday struggle (Moe & Ytterhus, 2022), adding to worries about the family's situation in the country of origin (Solhaug et al., 2024). When our participants lack recognition from primary relations, these needs must be acknowledged and met by others outside Honneth (2005) love form, placing additional demands on their primary contacts, as well as educational and health professionals. What these young people ask for is to be seen as persons when receiving health-related support and healthcare in everyday life and being recognized in their efforts to become active members of society, which, in Honneth terms, is a premise for becoming ordinary citizens (Honneth, 2005).

### 4.2. Meeting everyday needs of continuity and care

The young settled unaccompanied refugees expressed different experiences regarding the extent to which the three groups of professionals encountered in this study were able to meet their expectations and needs for caring relationships. They experienced school nurses and teachers as quite successful in providing care for them as whole persons. These professionals are trained within distinct disciplinary frameworks and are required to serve the population in accordance with national legal regulations. These differences may be partially attributed to the varying meeting frequencies and the different professionals' competencies and availability. The young participants met their teachers daily within the school setting, and the school nurses were generally accessible on a weekly basis, whereas consultation with their GPs often required booking days or weeks ahead.

Our results show that on an everyday basis, teachers constitute an important part of what we have called the trivial system in everyday life, and they are significant individuals when it comes to helping and supporting the young refugees. An earlier review showed that unaccompanied children and youths identified good relationships within care systems when professionals were experienced as interested, emotionally engaged, and attentive to their well-being (Kauhanen & Kaukko, 2020). In our study, teachers, primary contact persons, and school nurses were perceived as being on the right form. In the trivial everyday life, they are an important buffer for what is lacking in what Honneth refers to as the love form (Honneth, 2005). While Honneth's theory draws clear distinctions between different spheres and their corresponding forms of recognition, these results suggest that, in practice, such relations are more fluid, moving across and blurring the boundaries between spheres. Teachers can play a decisive role in supporting young refugees through their resettlement process. Pastoor (2014)found that teachers do not always recognize symptoms of the psychosocial and mental health needs of young unaccompanied refugees, but in our study, teachers were adults who provided continuity in the participants' everyday lives. They are not expected to solve young individuals' health issues, but through their presence and acknowledgement, they are perceived as people who care and provide support. The care and recognition that young unaccompanied refugees receive from teachers place the latter in a position between Honneth's love and rights forms.

In their healthcare profession, school nurses appear to occupy a space between the trivial and the special in the everyday lives of the young. Trivial is used here in the sense that they are available weekly free of charge, especially because the young unaccompanied refugees must initiate visits, and their health professional is not needed on a daily basis. However, since the participants are not used to this profession and area of healthcare, their expectations are limited. In a study on school nurses' experiences of working with refugee children and adolescents, one of the results was that school nurses considered it important to understand the other person's emotions and feelings (Musliu et al., 2019). This is consistent with the young unaccompanied refugees' experiences of the school nurses in the present study. The participants reported that they were able to talk to the nurse, who appeared as someone they could learn from in coping with everyday life. Being professional also entails being personal, an aspect that has also been shown to be important in patient‒doctor relationships (Arborelius & Bremberg, 1992).

### 4.3. The importance of recognizing the whole person in the clinical encounter

The relationship between the young unaccompanied refugees and GPs is different from their more frequent encounters with teachers or school nurses. GPs normally meet these young individuals when there are breaks in their trivial everyday life, an event that can be defined as special. The results from this study show that both system-related factors and personal factors influence participants' experiences of clinical encounters with GPs. First, the organizational structure of the GP differs in the sense that GPs typically work within the framework of a 15–20 min consultation (Hunskår, 2023, p. 59), unlike teachers and school nurses, who rarely operate under such strict time constraints. When the young unaccompanied refugees experienced that the GPs did not meet their expectations of availability, this contributed to a sense of lacking recognition. Experience of short consultation times and limited availability has also been reported among asylum seekers and refugees residing in the United Kingdom (Kang et al., 2019). Other frustrating system-related factors may include expectations of rapid access to healthcare services, unfamiliarity with the GP gatekeeping role, and restricted use of medications for minor problems, which are rooted in different normative understandings of health and medicine. These factors have also been reported as a challenge by GPs (Harris et al., 2020). To some extent, misalignment between expectations and realities might be mitigated by better knowledge of how the services are organized. At the same time, their young age may represent a resource, as—like other young people in transition to adulthood—they are still in the process of learning how to navigate health services. Our data illustrate that the young participants actively use their agency when experiencing health problems. They tried to manage on their own and reported that they consulted their GPs when they felt a genuine need for help and support. However, when young refugees seek care from their GPs, often with unspecific symptoms that may reflect a stressful life situation, the human need for recognition should be given more attention. This is not necessarily a structural challenge but rather one that arises within the interpersonal dynamics of the clinical encounter. In Honneth's framework of recognition, the role of the GP aligns with the legal relation, which is the right form. Although both GPs and school nurses operate within the rights form, school nurses are experienced as having a more care-based relationship. In contrast, the young unaccompanied refugees experienced that when the GPs did not understand their everyday life, they lacked both acceptance and recognition. These results align with previous research on migrants, including refugees, which highlights similar perceptions of unmet expectations in healthcare settings in other European countries, such as the Netherlands and the United Kingdom (Feldmann et al., 2007; Madden et al., 2017; Zijlstra et al., 2019). The importance of GPs knowing their patients as persons has been highlighted as challenging (Mjølstad et al., 2013). Previous research has also shown that many GPs find it difficult to determine whether and how to address a patient's history in situations where the patient appears likely to have experienced painful and adverse life events (Rønneberg et al., 2022). Existing studies (Flataas, 2021; Harris et al., 2020) describe that GPs felt that their education did not prepare them for working with refugees who suffer from mental health issues. At the same time, Kauhanen and Kaukko (2020) point out that recognition does not mean that all of an individual's desires and needs are satisfied, but rather that the expression of these needs is explicitly heard, acknowledged, and respected. This aligns with what Starfield (2011) called person-focused care, which is an extension of more narrowly defined patient-centered care. Person-focused care emphasizes that accumulated knowledge about the person serves as a foundation for understanding and recognizing health problems and needs, thereby facilitating appropriate care (Starfield, 2011).

GPs do not encounter young unaccompanied refugees as frequently as school nurses or teachers, which is natural given their different roles and service organization. Consequently, they have fewer opportunities to build trust and provide recognition than, for example, teachers. They also have less opportunity to correct an unfortunate consultation, as they are unlikely to meet the young person again the next day. However, this is not specific to unaccompanied refugees. Other groups of young people have also reported not being heard or recognized by their GPs. Adolescents with ill or substance-dependent parents have similarly expressed a lack of recognition from GPs, describing how they do not feel seen as a unique person in a demanding life situation when approaching their GP with relatively vague symptoms (Gullbrå et al., 2016). In the textbook description of the “clinical consultation” in general practice (Hunskår, 2023), it is, however, emphasized that GPs also need to understand the underlying factors behind the presented symptoms. This involves gaining insight into the reasons for the patient's visit, which is sometimes influenced by a challenging life situation and strained relationships. This may be especially challenging in relation to immigrant patients because of the complexity of their situation and language barriers (Radl-Karimi et al., 2020). However, the review by Radl-Karimi et al. (2020) highlights facilitating factors for co-productive relationships between immigrant patients and healthcare professionals, emphasizing that conveying the message *“I see you, I hear you, and I try to understand you”* is central for patients to feel respected and acknowledged. The results of our study indicate that GPs should strive to adopt such a relational approach, as it appears crucial for addressing the particular needs of young unaccompanied refugees.

Honneth argues that withdrawal or refusal of recognition may amount to personal harm (Honneth & Farrell, 1997). In this context, a GP's actions—whether shaped by choice, clinical routines, or time constraints—may lead young patients to feel inadequately acknowledged, with potential consequences for their self-respect and well-being. As Popovac (2022) emphasizes, healthcare is fundamentally about human decency and attending to the person behind the symptoms. When biomedical problems are evident, young patients often express satisfaction with GPs as competent medical experts. However, when symptoms are common and/or lack clear signs, clinically sound decisions not to pursue further investigation may be experienced as dismissive if not accompanied by a credible explanation or genuine interest in potentially underlying causes related to the patient's broader life context. For young unaccompanied refugees, such encounters may be perceived as a lack of care and recognition. In the absence of supportive primary relationships, person-centred care may help compensate by incorporating elements of Honneth's love form in addition to the legal dimension, thereby supporting self-confidence and self-respect (Honneth, 2005, p. 129) and preventing their identities from being undermined in everyday life (Gullestad, 1992). Conversely, failure to communicate such recognition may undermine both patients' trust and GPs' professional authority.

## 5. Strengths and limitations

A key strength of this phenomenological study lies in the completion of 19 in-depth interviews with young settled unaccompanied refugees—a challenge given the nature of the topic and the constraints imposed by the COVID-19 pandemic during the time of the interviews. Despite these obstacles, we consider our material to be rich and comprehensive. By applying Honneth (2005) as an analytical framework, this study offers nuanced insights into vertical relational aspects and highlights how the absence of recognition from primary relationships may adversely affect the self-esteem of these young individuals. Furthermore, the study gives voice to a small, vulnerable but also resourceful group of young people, contributing valuable perspectives to the development of a diverse society. Nevertheless, our study also has obvious limitations. As described in the methodology section, the participants declined the use of interpreters during the interviews because of their lack of trust in interpreters representing other political groups than themselves in their country of origin. While this decision may have constrained some of the nuance of their responses due to second-language use, it was respected based on the researchers' assessment of the participants' participation and the importance to foster an environment in which participants felt comfortable sharing their experiences, which might have been inhibited by the presence of an interpreter. Additionally, it is important to note that the study focuses on the experiences of the young unaccompanied refugees and does not account for the structural conditions under which primary-contact persons, teachers, school nurses, and GPs operate. There are discrepancies in existing research according to gender differences among unaccompanied minors' health and well-being, which might have been interesting to include with a higher number of participants.

## 6. Conclusion and implications

This paper highlights young, settled, unaccompanied refugees' need for help and support in the resettling process in Norway and the importance of recognition in developing a secure sense of self. Following Honneth (2005), healthy identity development relies on recognition in the loving, rights, and community-of-value forms, with deficits in one form potentially compensated through others. Most participants in our study lacked recognition in the loving form due to the absence of adult caregivers, which, combined with the reversed care roles of their parents, placed them at similar risk as children whose parents cannot fulfil societal expectations. Our results suggest that professionals who meet these young refugees on a regular or even occasional basis must actively acknowledge the recognition they lack in primary relationships. Such recognition is essential to support their self-confidence, everyday functioning, and health. In particular, GPs appear to have the potential to improve their engagement with this group. While addressing medical needs remains the core responsibility, person-centred care for young unaccompanied refugees should also involve recognizing both their vulnerabilities and strengths and creating space for their life experiences and concerns to be heard and valued.

## Data Availability

Anonymized data will be made available on reasonable request.
